# An osteoporotic bone model: developing and validating an ex-vivo bone demineralization protocol

**DOI:** 10.1093/jbmrpl/ziag069

**Published:** 2026-04-15

**Authors:** Fahad Alabdah, Adel Alshammari, Araida Hidalgo-Bastida, Glen Cooper

**Affiliations:** Department of Mechanical and Aerospace Engineering, School of Engineering, University of Manchester, Manchester, M13 9PL, United Kingdom; Mechanical Engineering Department, Engineering College, University of Ha'il, Ha'il, 2440, Saudi Arabia; Department of Mechanical and Aerospace Engineering, School of Engineering, University of Manchester, Manchester, M13 9PL, United Kingdom; Mechanical Engineering Department, Engineering College, University of Ha'il, Ha'il, 2440, Saudi Arabia; School of Biological and Chemical Sciences, Faculty of Science and Engineering, Manchester Metropolitan University, Manchester, M1 5GD, United Kingdom; Department of Mechanical and Aerospace Engineering, School of Engineering, University of Manchester, Manchester, M13 9PL, United Kingdom

**Keywords:** osteoporosis, mechanical properties, sheep bone, demineralization, ex vivo model

## Abstract

Studies estimate that there are 500 million osteoporotic patients worldwide. Osteoporosis leads to 2.7 million hip fractures annually. These fractures usually require synthetic implants known as total hip replacement. Orthopedic implant development is an ongoing process, which requires numerous tests to evaluate their feasibility, in addition to the International Standards Organization regulatory tests. These tests require human cadaveric bones and animal in vivo experiments, which are associated with high costs, are time-consuming and limited through regulations, which poses a challenge. Following the replacement, reduction and refinement principle in animal testing (3Rs), this study aims to develop and evaluate a protocol where osteoporotic samples are prepared through ex vivo procedure using sheep bones from the food chain, given their availability and size, which is considerably similar to human cadaveric bones. Sheep femurs were demineralized using hydrochloric acid at different time intervals, resulting in changes in the bone architecture, mineral density, and mechanical properties. Demineralized specimens exhibited progressive reductions in mechanical performance and trabecular integrity with increasing demineralization time. Young’s modulus decreased from 110.7 ± 28.8 MPa in Control group to 88.7 ± 16.0 MPa and 57.7 ± 6.0 MPa in the 48H and 96H groups, respectively, accompanied by corresponding reductions in failure load. Trabecular microstructure was significantly altered, with trabecular thickness decreasing and trabecular separation and porosity increasing, reaching an approximately 30% higher porosity in the 96H group compared with Control group. Volumetric BMD (vBMD) was significantly reduced by both demineralization treatments (33% relative decrease). Despite the limited sample size, consistent trends indicate meaningful changes with demineralization. The observed structural and mechanical properties fall within reported ranges for human osteoporotic trabecular bone, supporting the use of demineralized sheep bone as a cost-effective ex vivo model for osteoporotic studies and preliminary orthopedic device evaluation.

## Graphical Abstract

**Graphical Abstract:**
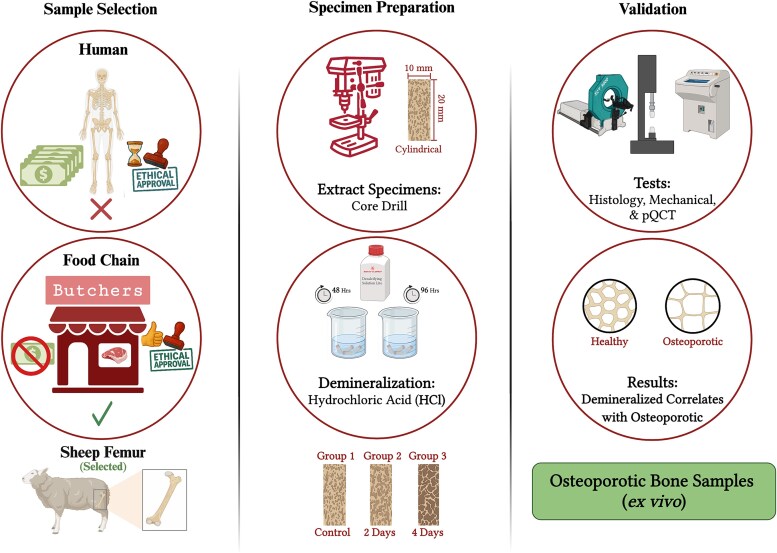

## Introduction

Osteoporosis has been estimated to affect 500 million people over the age of 50 worldwide.^1^ Women are at greater risk of being affected by osteoporosis compared to men, as it has been reported that 21.2% of postmenopausal women are osteoporotic compared to 6.3% in men,^2^ due to reduced estrogen levels. This skeletal condition leads to 2.7 million hip joint fractures annually worldwide, which leads to a mortality rate of 20%-40% in the first year after fracture occurrence.^3^ Treatment for hip fractures is a surgical intervention known as total hip replacement (THR), which despite being the standard clinical procedure for hip fracture it still has a mortality rate of 1.2%,^4^ with many patients reporting severe pain from both the hip fractures and post procedure. Additionally, there are approximately 90 000 THR procedures per year,^5^ which cost £4.4 billion per year in the UK.^6^ The surgical revision rate was high in 2018 at 6.6% of the total number of THR performed, due to clinical complications, such as aseptic loosening, infection, periprosthetic fractures, and adverse reaction to particulate debris.^5^ The nature of revision surgery is more complex considering the condition of the existing implant, and the time and cost associated with it.^7^ Therefore, osteoporosis is problematic and requires novel treatment approaches to reduce the catastrophic clinical complications, limited quality of life for patients, and high financial costs.

The process of introducing an orthopedic device such as joint arthroplasty often involves evaluation and assessment of that device using cadaveric bones prior to in vivo experiments (preclinical) and clinical trials. This process usually requires many samples due to the variation of bone quality including differences based on sex, age, ethnicity, existence of skeletal diseases, and the stage in which those diseases are in. The need of a large sample size of cadaveric bones means high procurement cost, and limited availability especially with the presence of diseases, such as osteoporosis, and even after numerous samples there will still be batch to batch variation.^8^

Synthetic femurs have been used for the purposes of assessment and reported to be a reliable representation.^9^ These femurs represent healthy bone samples with less variation in comparison with cadaveric ones. A synthetic femur that represents osteoporotic cadaveric bone was introduced by Gluek et al. and reported to show acceptable imitation of the osteoporotic condition.^10^ However, it has not been commercialized yet. In addition, natural tissue would show better biocompatibility compared to synthetic tissues as they would have statistical variation and display failure modes similar to bio-composite bone tissue.

It is hypothesized that demineralized sheep bone from the food chain will be a good model for osteoporotic human bone. The aim of this study is to develop and evaluate a procedure where osteoporotic samples are prepared ex-vivo using sheep bone samples from the food chain, given their availability and size, which are considerably similar to human cadaveric bones. There are advantages to substituting cadaveric samples for biomedical research, which would eliminate the high procurement costs, and negate the time-consuming regulations associated with the supply of human osteoporotic specimens, which makes ex vivo animal tissue and in silico experiments attractive approaches. This would also enhance the implementation of the (3Rs) principal in biomedical research,^11^ which states that in vivo experiments should be replaced, reduced or refined where possible. Therefore, sheep bone samples were demineralized in this study to represent osteoporotic conditions using decalcification solutions. Mechanical testing, histology, and pQCT images were performed to evaluate the validity of the protocol through mechanical properties, architecture and mineral density of the bone.

## Materials and methods

### Sample preparation and storing

**Figure 1:**
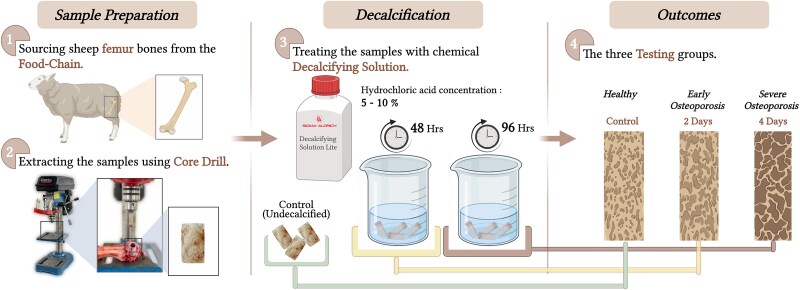
Schematic illustration of the ex vivo osteoporotic bone model preparation process. Created with BioRender.com.

The bones used to extract the samples have been sourced from the food chain which is discarded tissue. Sheep bones were sourced from a butcher (The Butcher’s Quarter), which supplies meat fit for human consumption. The samples were prepared immediately on arrival at the laboratory using a core drill (Clarke CDP5EB 5 Speed Bench Mounted Pillar Drill with 10 mm core drill bit) as shown in Figure 1. The shape of the samples was cylindrical with 8 mm diameter and 16 mm high. The samples were frozen in phosphate-buffer saline (PBS) at −20 °C as it has been reported to be a sufficient processing method that maintains the samples’ stiffness.^12,13^ The samples were left at 4 °C overnight to defrost before assessment. Samples were prepared and divided into 3 groups; each test was repeated at least 3 times.

### Decalcification of bone samples

Samples were treated with 0.5 mL of Decalcifying Solution-Lite (Sigma-Aldrich Solutions), which is a hydrochloric acid (HCl), with a concentration range of 5% to less than 10%. Hydrochloric acid was employed for bone demineralization as it reproduces the fundamental chemical mechanism underlying osteoclastic mineral resorption, namely proton-driven dissolution of the bone mineral phase. Mammalian bone mineral is primarily composed of a non-stoichiometric, carbonated, calcium-deficient form of hydroxyapatite, which exhibits enhanced solubility under acidic conditions compared with geological apatites.^14^ During physiological bone remodeling, osteoclasts establish a localized acidic environment that facilitates mineral dissolution through protonation of hydroxyl and phosphate groups within the hydroxyapatite lattice.^15^ Treatment of bone with HCl in vitro induces a comparable chemical process, resulting in the net dissolution of hydroxyapatite, which can be represented by the following stoichiometric reaction (eqn 1):


1 \begin{eqnarray*} \mathrm{C}{\mathrm{a}}_{10}{\left(\mathrm{P}{\mathrm{O}}_4\right)}_6{\left(\mathrm{OH}\right)}_2+20\mathrm{HCl}\to 10\mathrm{CaC}{\mathrm{l}}_2+6{\mathrm{H}}_3\mathrm{P}{\mathrm{O}}_4+2{\mathrm{H}}_2\mathrm{O}. \end{eqnarray*}

Equation 1 provides a simplified representation of the overall chemical outcome of acid-mediated demineralization based on the known composition and solubility behavior of bone mineral.^14^ It is a proton-mediated hydroxyapatite dissolution, the same fundamental process underlying osteoclastic mineral resorption.

The treatment was performed at room temperature, and the samples were washed with deionized water before and after the treatment. Demineralization progress was monitored using pQCT by measuring changes in BMD during acid treatment to verify consistent removal of the inorganic phase across specimens. The demineralized samples were divided into 2 groups, one group was left in solution for 48 h (48H Group) and the other group 96 h (96H Group). A third group of untreated samples was set as Control group.

### Mechanical axial compression tests

**Figure 2:**
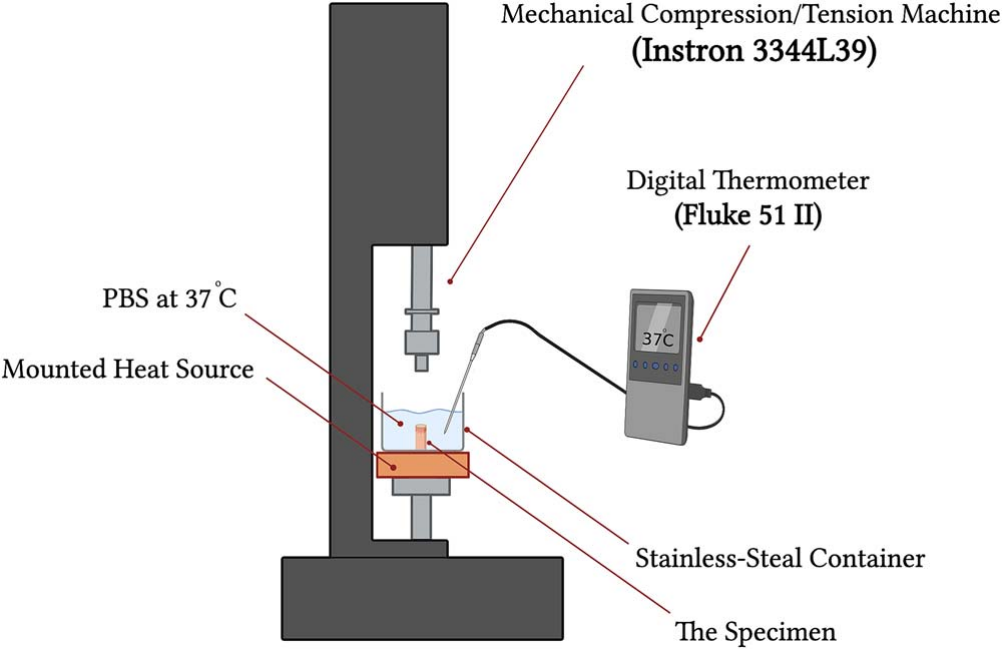
Schematic illustration of the mechanical testing setup. Created with BioRender.com.

The compressive failure load and stiffness of control and demineralized samples were measured through quasi-static loading. Compressive stiffness and failure loads were measured by placing the samples between 2 compression plates on a mechanical test machine (Instron 3344L39), as shown in Figure 2. The mechanical test machine was fitted with a 1 kN load cell, and the test machine had recently been calibrated and serviced by the manufacturer. Each sample was cut on both ends using a V block to make sure that all surfaces were flat to avoid any misalignment or slippage during the test. The samples were tested at a displacement-controlled loading rate of 0.5 mm/min, consistent with quasi-static compression protocols commonly used for trabecular bone testing in the literature,^16,17^ and they were placed in PBS at 37 °C to imitate the physical environment inside the human body during the test.^18^ The tests were carried out using a stainless-steel container to endure the loading and maintain the temperature of the PBS at 37 °C through the heat source due to its thermal conductivity. The temperature was measured using a Fluke 51 II single input digital thermometer (Fluke Corporation), during the testing. The biomechanical parameters Young’s modulus (MPa), yield stress (MPa), and failure load (N), were assessed for each group after the data for all specimens was collected. The typical force–displacement and stress–strain curves were obtained. The Young’s modulus and yield stress were obtained from the stress–strain curve, where the liner region was used to calculate the Young’s modulus, and the yield stress was defined as the stress at which the trabecular structure commences to deform plastically. Trabecular cores were tested in their extracted orientation without alignment to the principal trabecular fabric; therefore, the reported Young’s modulus represent apparent, orientation-averaged stiffness rather than direction-specific (longitudinal or transverse) properties. The failure load was obtained from the force–displacement curve where the compression force decreased as a result of the additional compressive increment force on the trabecular specimen, which led to bone failure.

### Histology

**Figure 3:**
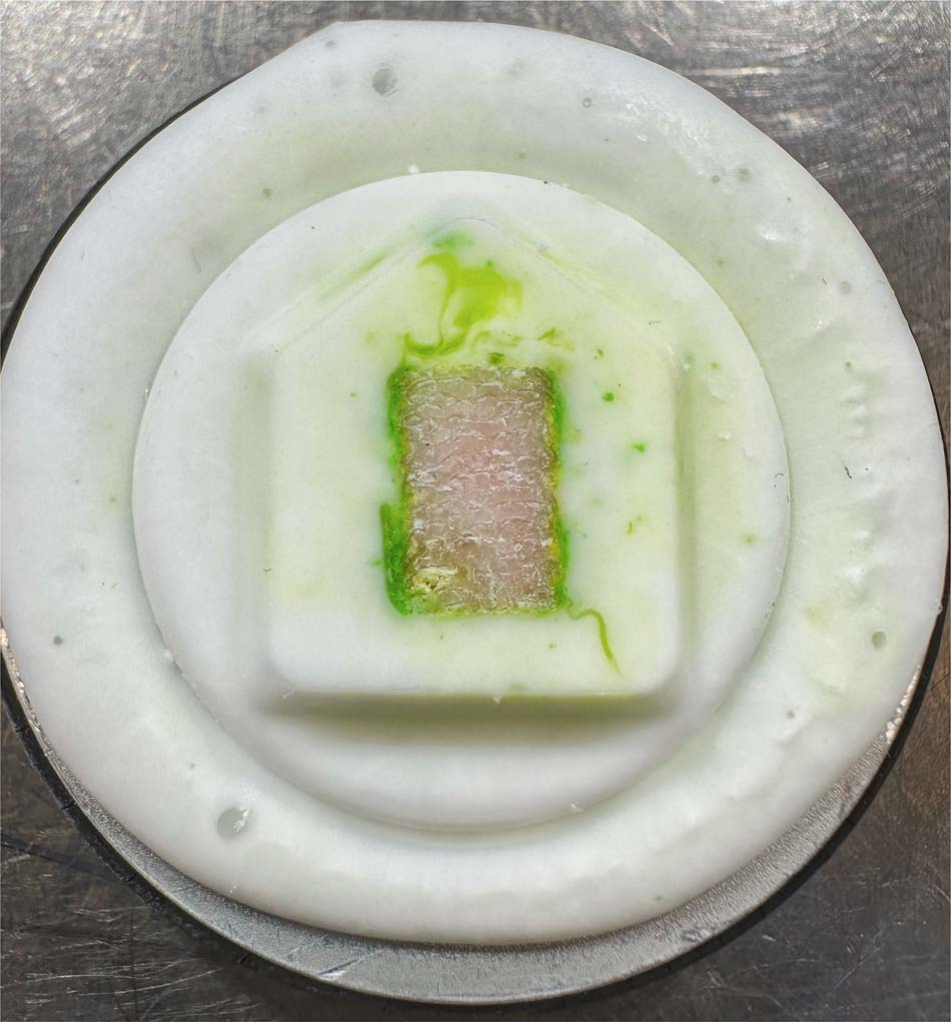
Bone sample embedded in optimum cutting temperature (OCT) compound during sectioning.

The architecture of the trabecular samples had to be evaluated to observe the changes in the porosity. Therefore, the samples were sectioned using a Leica CM1950 cryostat (Leica Biosystems). Optimum cutting temperature (OCT) compound was used as an embedding medium for the specimens which rapidly frozen, then sectioned at 10 μm thickness and −30 °C as shown in Figure 3. The slices were attached to microscope slides and imaged using a Leica DMi1 inverted microscope (Leica Microsystems). The acquired images were converted to binary format and analyzed to quantify trabecular morphology. In addition to porosity, trabecular thickness (Tb.Th) and trabecular separation (Tb.Sp) were calculated using 2-dimensional image-analysis tools in ImageJ (National Institutes of Health). These parameters were derived from the binarized images using distance-based algorithms that estimate local thickness and spacing across the trabecular network. Because the analysis was based on 2-dimensional images rather than micro-CT volumes, connectivity-based and 3-dimensional architectural parameters could not be reliably determined and were therefore not evaluated.

### Peripheral quantitative computed tomography

Peripheral quantitative computed tomography was used to analyze the differences in volumetric bone density in a quantitative manner to evaluate the impact of the demineralization process. A Stratec XCT3000 pQCT scanner (Stratec Medizintechnik GmbH), which was recently serviced by the manufacturer, at a resolution of 0.8 mm voxel size. All tests were performed at room temperature. The data extracted provided volumetric BMD (vBMD) values in milligrams per cubic centimeter ($\mathrm{mg}/{\mathrm{cm}}^3$).

### Statistical analysis

All quantitative outcomes are presented as mean ± SD. Each experimental group (Control, 48H, and 96H) consisted of 3 biological replicates (*n* = 3 per group). Group differences were evaluated using one-way analysis of variance (ANOVA). When ANOVA indicated a significant or near-significant group effect, Tukey’s honestly significant difference (HSD) test was used for post-hoc pairwise comparisons. For Young’s modulus, the effect size was quantified using eta-squared (η^2^) to account for the limited sample size. Statistical significance was defined as *p* < .05, with trends and effect sizes reported where appropriate to capture biological differences.

## Results

### Mechanical axial compression tests

**Figure 4:**
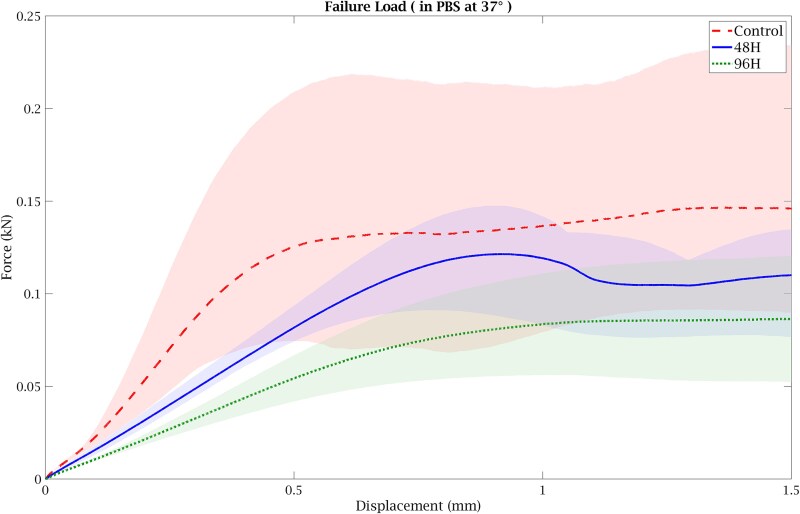
The force–displacement curve of the averaged control and demineralized bone samples (3 samples each condition).

**Figure 5:**
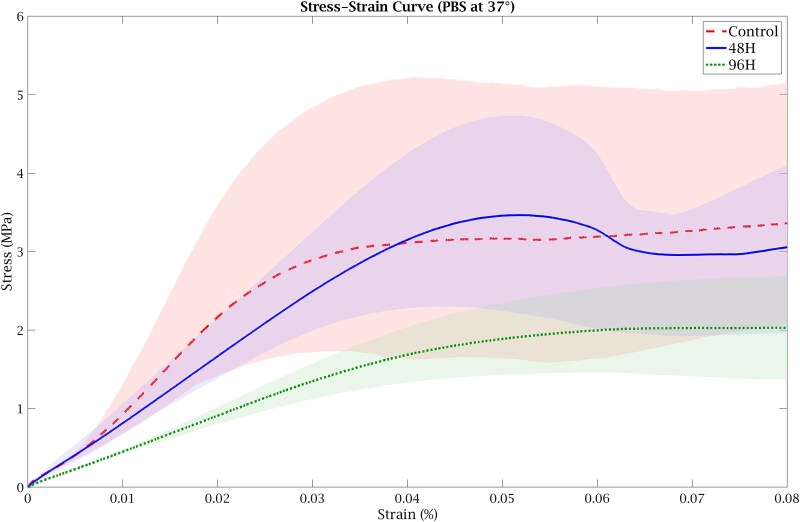
The stress–strain curve of the averaged control and demineralized bone samples (3 samples each condition).

Failure load, determined as the peak force from the force–displacement curves (Figure 4), decreased from Control group (128.2 ± 72.7 N) to 48H group (110.3 ± 30.3 N) and 96H group (78.2 ± 29.9 N). One-way ANOVA did not demonstrate a statistically significant group effect (F(2,6) = 0.87, *p* = .464). The corresponding effect size was η^2^ = 0.23, indicating that a portion of the variance in failure load was attributable to group differences. Analysis of the stress–strain curves (Figure 5) showed a reduction in Young’s modulus with increasing demineralization duration. Modulus values were 110.7 ± 28.8 MPa in Control group, 88.7 ± 16.0 MPa in 48H group, and 57.7 ± 6.0 MPa in 96H group. One-way ANOVA revealed a significant group effect (F(2,6) = 6.03, *p* = .036; η^2^ = 0.67). Post-hoc Tukey testing indicated a significant reduction in modulus in the 96H group compared to Control group (*p* = .034). Yield stress followed a similar decreasing pattern (Control group: 3.03 ± 1.61 MPa; 48H group: 2.65 ± 0.65 MPa; 96H group: 1.75 ± 0.32 MPa) (Figure 5). Although the group effect did not reach statistical significance (F(2,6) = 2.68, *p* = .148), the effect size (η^2^ = 0.47) suggests variability between groups. Overall, these results demonstrate reductions in trabecular bone mechanical properties with increasing demineralization duration, most evident in the 96H group. All biomechanical markers evaluated in this study showed lower mechanical properties in the demineralized groups compared to the control group. The 96H group results have similar trends in mechanical competence reduction to those reported in human osteoporotic bones as shown in Table 1.

**Table 1:** Comparison between the mechanical properties of human osteoporotic bone samples and demineralized sheep bone samples.

Biomedical marker	Human (osteoporotic) (*n* = 28)	Sheep (demineralized) (96H Group) (*n* = 3)
**Young’s Modulus (MPa)**	60.71 ± 3.03^23^	57.7 ± 6.0
**Yield stress (MPa)**	1.94 ± 1.2^23^	1.75 ± 0.32

### Histology

**Figure 6:**
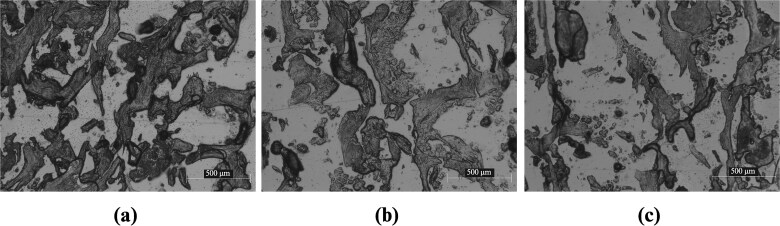
Microscope images of the slices showing the trabecular structure: (a) control, (b) 48H, and (c) 96H, (darker color) bone structure (lighter color) voids.

The cryotome sectioning has revealed slices showing the structures of the samples to observe the porosity and architecture. It can be clearly seen that demineralized samples are more porous and have a less dense architecture. The slices were observed under the optical microscope as shown in Figure 6 and the porosity of each sample has been quantified through pixel count from binary images. Marked specimen-dependent alterations in trabecular structure were observed. Tb.Th decreased from Control group (157.4 ± 13.4 μm) to 48H group (126.7 ± 3.7 μm) and 96H group (90.9 ± 7.0 μm), while Tb.Sp increased from 314.3 ± 9.8 μm (Control group) to 364.8 ± 29.2 μm (48H group) and 418.5 ± 8.8 μm (96H group). One-way ANOVA showed highly significant group effects for both Tb.Th (F(2,6) = 41.32, *p* = .00031) and Tb.Sp (F(2,6) = 23.77, *p* = .00141). Tukey post-hoc testing demonstrated that all pairwise comparisons (Control: 48H, Control: 96H, and 48H: 96H) were significant for both parameters indicating progressive trabecular thinning and increasing separation with specimen complexity.

Trabecular porosity increased monotonically from Control group (0.506 ± 0.046) to 48H group (0.574 ± 0.019) and 96H group (0.660 ± 0.051). One-way ANOVA confirmed a significant group effect (F(2,6) = 10.46, *p* = .011). Tukey post-hoc analysis showed that 96H group specimens produced significantly higher porosity than Control group (*p* = .009), corresponding to an approximately 30% relative increase, while the 48H group showed an intermediate, non-significant increase.

### Peripheral quantitative computed tomography

**Figure 7:**
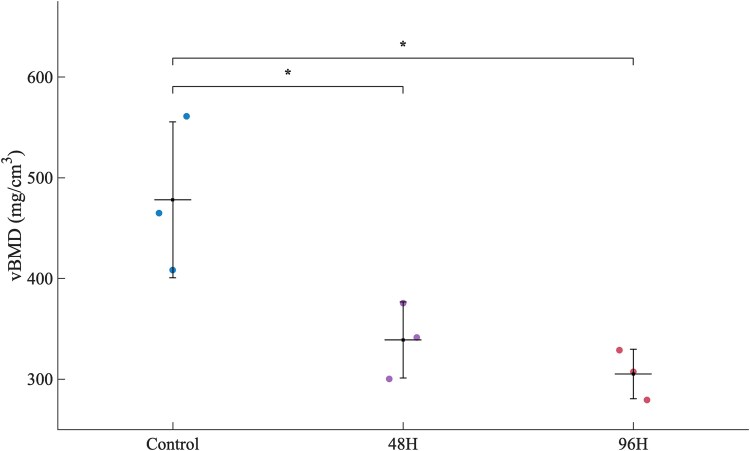
The volumetric BMD (vBMD) of the samples extracted from Peripheral quantitative computed tomography (pQCT), showing the significance between the control and demineralized groups, *p* < .05.

The results of the pQCT scanning revealed significant variations in vBMD between the control, 48H, and 96H groups as shown in Figure 7. Trabecular vBMD was significantly reduced by both specimen treatments (Control group: 478.1 ± 81.6 mg/cm^3^; 48H group: 336.7 ± 49.1 mg/cm^3^; 96H group: 325.3 ± 7.6 mg/cm^3^). One-way ANOVA showed a significant effect of group (F(2,6) = 7.15, *p* = .0259). Tukey post-hoc testing indicated that both 48H group (*p* = .0458) and 96H group (*p* = .0337) had significantly lower vBMD than Control group, while no difference was detected between 48H group and 96H group. These findings highlight the effects of the experimental settings on bone density, as seen by the 33% decrease in vBMD in the 96H group compared to Control group. These outcomes have a similar trend with the drop in vBMD in human osteoporotic patients which is 34% compared to the healthy population.^19^

## Discussion

This study hypothesized that osteoporotic bone samples could be developed using an ex vivo approach to reduce orthopedic device testing costs and time. The protocol developed in this study has achieved the establishment of osteoporotic conditions in trabecular structure in animal bones from the food chain. This approach significantly reduces the cost of the testing since the bones could be acquired from the food chain for no cost. The significance of the cost reduction is in comparison to the procurement of human cadaveric osteoporotic bones and the in vivo animal studies to establish osteoporosis. This protocol also reduces the time of developing the osteoporotic samples, considering the ethical approvals associated with the handling of human tissue, or the time for in vivo osteoporosis inducement. The results of the mechanical properties, architectural changes, and BMD in this study have been shown to correlate well with the data from data in the literature on human osteoporotic bones and in vivo data for animal osteoporotic models, which validates the protocol for the ex vivo establishment of osteoporotic bone samples.

There are some studies that utilized the demineralization of animal bones to establish osteoporotic conditions; however, these have focused on the cortical part which led to the removal of the trabecular part and the bone marrow,^20^ or utilized vertebrae bone.^21,22^ However, the protocol developed in this study focuses on the establishment of osteoporosis in the trabecular bone structure in the femur. The protocol was assessed by analyzing the changes in trabecular mechanical properties, architecture, and vBMD.

The mechanical properties of the demineralized samples were evaluated by their elasticity using the young’s modulus and yield stress as markers. It has been shown have similar trends as human osteoporotic bone reported by Ozan et al.,^23^ after 96 h of demineralization. In addition, the vBMD of those specimens was found to be 33% less than the control group, which also aligns with the relative change in vBMD between healthy and osteoporotic bone samples, which is 34%.^19^ Moreover, the relative increase in trabecular porosity was found to be 30%. These differences correlate exactly with the in vivo data of the trabecular structure of sham and ovariectomized sheep samples, which is 30%.^24^

The development of ex vivo models could also impact the outcomes of in silico clinical experiments. The recent investigations that address biomedical challenges have been implementing in silico techniques.^25,26^ Although in silico studies fulfill the three Rs principle, they need data from experimental studies for the development of the models and validation. This ex vivo protocol, however, could provide data to be reverse engineered in order to enhance the accuracy and validity of the simulations.^27–30^ The cost-effectiveness, the low time consumption, and the alignment of this protocol with the 3 Rs principle make it an invaluable tool for biomedical research.

Several limitations of this study should be acknowledged. Although the demineralized specimens do not reproduce the absolute values of human trabecular bone in all measured parameters, including mineral density, porosity, and mechanical stiffness, the relative trends observed between control and demineralized groups are consistent with those reported for the transition from healthy to osteoporotic bone. Differences in species, bone size, geometry, and trabecular architecture between ovine and human femora inevitably contribute to quantitative discrepancies. In addition, the extent of demineralization that can be applied to sheep trabecular bone is constrained by the need to preserve structural integrity, as excessive mineral removal leads to collapse of the trabecular network and a disproportionate loss of mechanical strength. While the limited sample size (*n* = 3 per condition) means that the results are not statistically significant they do indicate that this may be a good model. This study was exploratory experiment which showed that the consistent trends observed are indicative that the demineralized specimens provide a meaningful model of osteoporotic bone. Within these biological and methodological constraints, the protocol produces reproducible reductions in mineralization, microstructural integrity, and mechanical performance that fall within reported ranges for osteoporotic bone, supporting its use as a controlled and indicative experimental model rather than a direct replica of the human condition.

Given the well-established similarities between human osteoporotic bone and ovariectomized sheep models reported in the literature, the ex vivo demineralization approach described here may help to reduce reliance on in vivo experiments, thereby offering ethical, cost, and time advantages for the preliminary evaluation of orthopedic devices.

## Data Availability

The data supporting the findings of this study are available within the article. Additional details are available from the corresponding author upon reasonable request.
